# Profiles of microRNA in aqueous humor of normal tension glaucoma patients using RNA sequencing

**DOI:** 10.1038/s41598-021-98278-0

**Published:** 2021-09-24

**Authors:** Hyemin Seong, Hyun-kyung Cho, Changwon Kee, Dae Hyun Song, Min-Chul Cho, Sang Soo Kang

**Affiliations:** 1 Department of Anatomy and Convergence Medical Science, Gyeongsang National University, Jinju, Republic of Korea; 2 Department of Ophthalmology, Gyeongsang National University Changwon Hospital, School of Medicine, Gyeongsang National University, Changwon, Republic of Korea; 3 Institute of Health Sciences, School of Medicine, Gyeongsang National University, Jinju, Republic of Korea; 4 Department of Ophthalmology, Samsung Medical Center, Sungkyunkwan University School of Medicine, Seoul, Republic of Korea; 5 Department of Pathology, Gyeongsang National University Changwon Hospital, Changwon, Republic of Korea; 6 Department of Laboratory Medicine, Gyeongsang National University Hospital, Gyeongsang National University College of Medicine, Jinju, Republic of Korea

**Keywords:** Bioinformatics, Gene expression analysis, Sequencing, Optic nerve diseases, Clinical genetics, Disease genetics, RNA sequencing

## Abstract

We aimed to identify and compare microRNAs (miRNAs) from individual aqueous humor samples between normal-tension glaucoma (NTG) patients and normal controls. Aqueous humor (80 to 120 µl) was collected before cataract surgery. Six stable NTG patients and seven age-matched controls were included in the final analysis. RNA sequencing was conducted for RNA samples extracted from the 13 aqueous humor samples, and bioinformatics analysis was employed for the miRNA targets and related pathways. Two hundred and twenty-eight discrete miRNAs were detected in the aqueous humor and consistently expressed in all samples. Eight significantly upregulated miRNAs were found in the NTG patients compared to the controls (fold-change > 2, *p* < 0.05). They were hsa-let-7a-5p, hsa-let-7c-5p, hsa-let-7f-5p, hsa-miR-192-5p, hsa-miR-10a-5p, hsa-miR-10b-5p, hsa-miR-375, and hsa-miR-143-3p. These miRNAs were predicted to be associated with the biological processes of apoptosis, autophagy, neurogenesis, and aging in the gene ontology categories. The related Kyoto encyclopedia of genes and genomes pathways were extracellular matrix-receptor interaction, mucin-type O-glycan biosynthesis, biotin metabolism, and signaling pathways regulating the pluripotency of stem cells. The differentially expressed miRNA in the NTG samples compared to the controls suggest the possible roles of miRNA in the pathogenesis of NTG. The underlying miRNA-associated pathways further imply novel targets for the pathogenesis of NTG.

## Introduction

Glaucoma is one of main public health problems and the second most common cause of visual impairment in the world that can lead to blindness^1^. It is estimated that there will be 79.6 million patients with glaucoma by 2020 and currently, Asians comprise half (47%) of the patients with glaucoma^1^. Glaucoma is characterized by a progressive injury to the optic disc followed by loss of the retinal nerve fiber layer (RNFL) and corresponding visual field defects^2^. Glaucoma is a neurodegenerative disorder and the pathological feature is death of retinal ganglion cell (RGC)^2,3^. Glaucoma is recognized as a multifactorial optic neuropathy and remains a disease of unknown etiology. Intraocular pressure (IOP) elevation is the main risk factor of glaucoma. However, risk factors other than IOP, such as vascular injury and hypoxia, are also related with the pathogenesis of glaucoma^3–5^. Primary vascular dysregulation (PVD) has been recognized as an important risk factor, especially for normal-tension glaucoma (NTG)^6–8^. The prevalence of NTG is higher in Asians than in other ethnicities, which is a distinctive feature^9^.

The difference in the type of glaucoma among ethnicities suggests that genetic factors are important in the development of glaucoma. Moreover, family history of glaucoma is widely recognized as one of risk factors for glaucoma. Thus, genetic background is considered to contribute to the occurrence of glaucoma and its pathogenesis^10–12^. Several genes have been demonstrated to be related with primary glaucoma, for example, optineurin (*OPTN*), myocilin (*MYOC*), optic atrophy 1, and neurotrophin 4^13–17^. However, these genes account for only up to 5% of the primary open-angle glaucoma (POAG) patients^18^ and have incomplete and low penetrance for the disease.

MicroRNAs are noncoding small (~ 18 to 22 nt) oligoribonucleotides that regulate the post-transcriptional modulation of gene expression via the perception of particular sequences in target mRNAs^19^. These microRNAs function principally to diminish the expression of target gene^20^. Contrary to mRNAs, miRNAs demonstrate notable stability within biofluids^21^. Aqueous humor has been suggested to have potential molecular biomarkers with significant pathophysiologic relevance^22^. Previous studies have identified microRNAs in the aqueous humor of normal subjects and POAG patients^23–26^. However, no previous study has evaluated microRNA expression in the aqueous humor of NTG patients. The pathogenesis and mechanism of development of NTG remain unknown.

Preliminary studies pooled aqueous humor samples to obtain enough volume for analysis. However, in the present study, we employed RNA sequencing to analyze the microRNA expression in individual aqueous humor samples. To our knowledge, the profile and expression of microRNA in aqueous humor from patients with NTG have not yet been reported. In the current study, we investigated the expression of microRNA in the aqueous humor of NTG patients compared to normal controls using RNA sequencing in a single ethnic group of Asians. Differentially expressed microRNAs may suggest a clue to the pathogenesis of NTG, which has an IOP in the normal range and is especially prevalent in Asians.

## Results

### Baseline characteristics and demographics of subjects

After library preparation and the quality check test, six NTG patients and seven age-matched control subjects were included in the final RNA sequencing. The baseline characteristics of the included NTG and control group subjects are shown in Table 1. The mean age of the NTG subjects (n = 6) and the control subjects (n = 7) was 65.5 ± 10.6 years and 63.9 ± 9.9 years, respectively. The mean IOP of the NTG and the control group was 14.8 ± 1.8 mmHg and 15.43 ± 2.3 mmHg, respectively. Each NTG patient was using just one topical medication, including fixed combination eye drops. The included subjects had no other ocular comorbidity than simple cataracts.

**Table 1:** Baseline characteristics and demographics of subjects.

Subject number	Disease status	Age, yr	Sex	Eye Laterality	Mean IOP, mm Hg	Topical Medication	Ocular comorbidity
1	Control	75	Female	Left	14	None	None
2	Control	57	Female	Right	14	None	None
3	Control	76	Female	Left	12	None	None
4	Control	54	Male	Right	16	None	None
5	Control	53	Male	Right	16	None	None
6	Control	71	Female	Right	17	None	None
7	Control	61	Male	Right	19	None	None
8	NTG	56	Female	Left	13	Latanoprost	None
9	NTG	57	Male	Left	13	Latanoprost	None
10	NTG	55	Male	Left	17	Dorzolamide/timolol	None
11	NTG	77	Female	Right	14	Dorzolamide/timolol	None
12	NTG	72	Female	Right	15	Latanoprost	None
13	NTG	76	Female	Right	17	Tafluprost	None

*IOP* intraocular pressure, *NTG* normal-tension glaucoma.

### Differential miRNA expression in aqueous humor from normal-tension glaucoma using RNA sequencing

**Figure 1:**
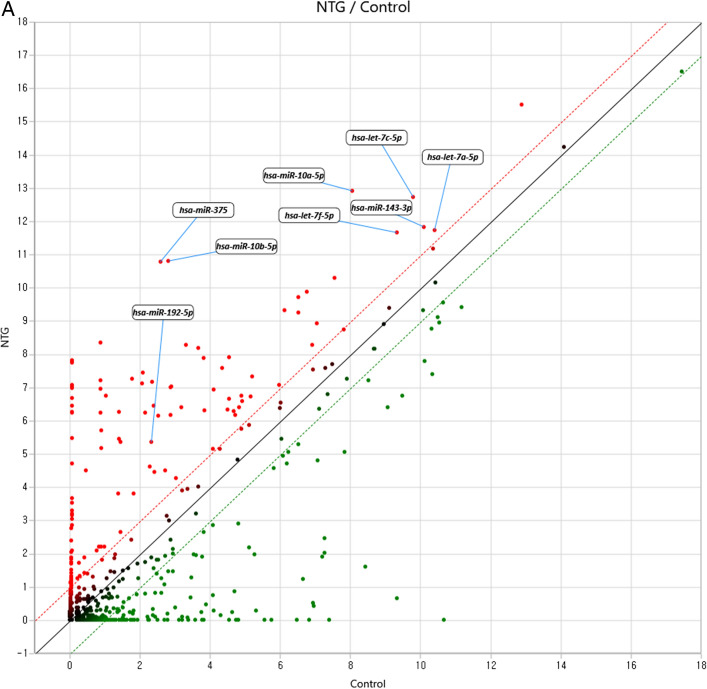

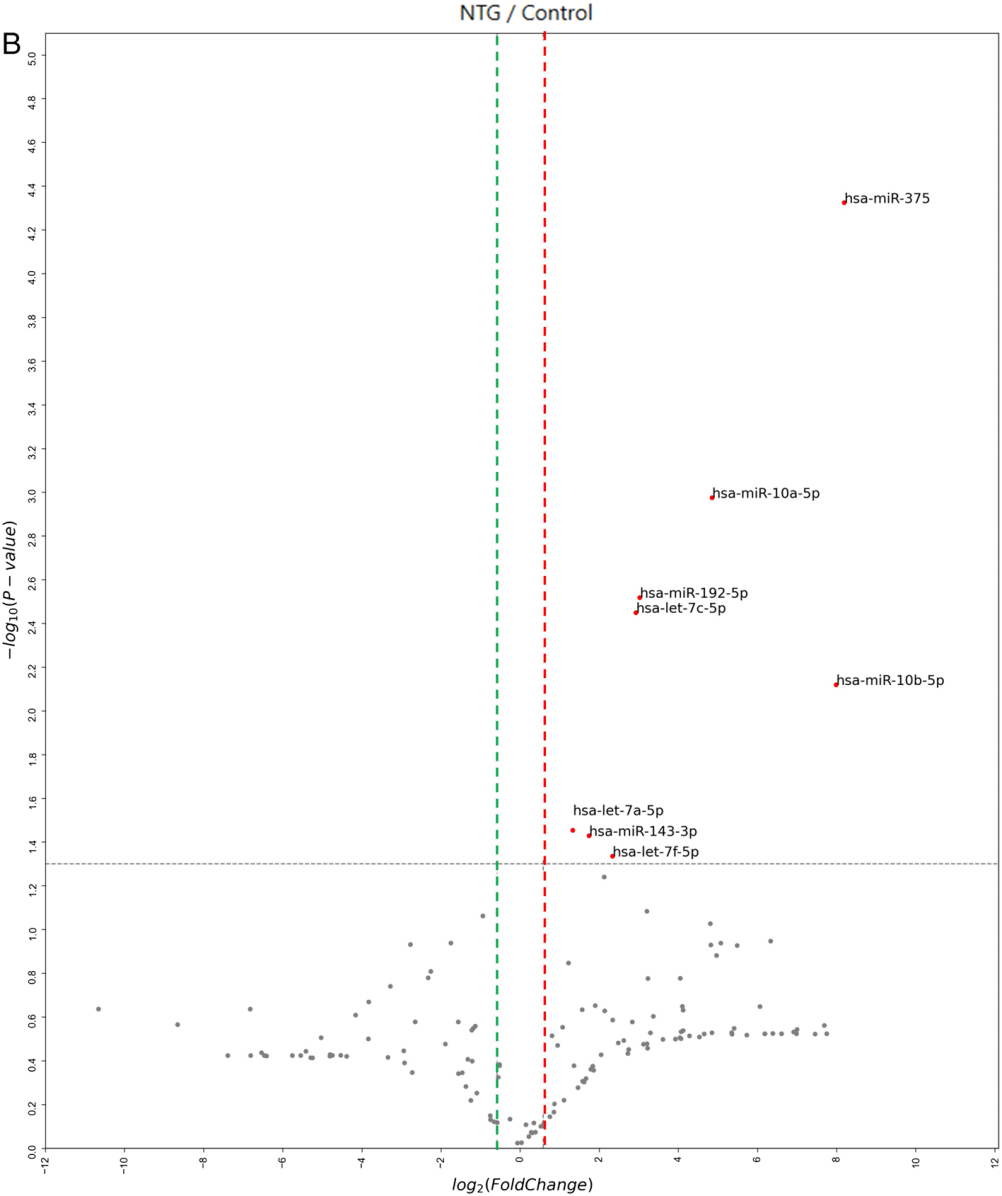
Scatter plot and volcano plot of the comparison of miRNA expression in aqueous humor from subjects with NTG versus unaffected controls. (**A**) Up-regulated miRNAs are shown to the upper region of the scatter plot (red) and down-regulated miRNAs are shown to the lower region of the scatter plot (green). A total of 228 mature miRNAs were identified in the aqueous humor of the NTG patients. Eight miRNAs, hsa-let-7a-5p, hsa-let-7c-5p, hsa-let-7f-5p, hsa-miR-192-5p, hsa-miR-10a-5p, hsa-miR-10b-5p, hsa-miR-375, and hsa-miR-143-3p were significantly up-regulated in NTG group compared to normal control. Fold change > 2 (red dotted line), fold change <  − 2 (green dotted line); Normalized data (log2) > 4; *p*-value < 0.05 versus control. (**B**) In volcano plot, up-regulated miRNAs are shown to the right of the plot (red) and were only selected which passed the thresholds of *p* < 0.05 (horizontal black dotted line) and fold change > 2 (red vertical dotted line). Down-regulated miRNAs are shown to the left of the plot (green) and were only selected which passed the thresholds of *p* < 0.05 (horizontal black dotted line) and fold change < 2 (green vertical dotted line). After selecting only those with Normalized data (log2) > 4, the volcano plot shows eight significantly upregulated miRNAs with fold change > 2, and *p*-value < 0.05. Of these, eight miRNAs were significantly up-regulated compared to the controls (fold-change > 2, *p* < 0.05), in addition to the analysis of the fold-changes were selected which only based on the normalized array data (log2) > 4. There was no significantly down-regulated miRNAs identified in NTG patients compared to control subjects according to the same significance criteria. Plots were presented by ExDEGA v1.2.1.0 software. *NTG* normal tension glaucoma.

The miRNA targets were analyzed based on the data from miRWalk 2.0. 2588 miRNAs were tested by RNA sequencing. And a total of 228 mature miRNAs were identified in the aqueous humor (AH) of the NTG patients (Fig. 1A). Up-regulated miRNAs are shown to the upper region of the plot (red) and down-regulated miRNAs are shown to the lower region of the plot (green). Of these, 8 miRNAs were significantly up-regulated compared to the controls (fold-change > 2 or < -2, *p* < 0.05), in addition to the analysis of the fold-changes were selected based on the normalized array data (log2) > 4 (Fig. 1B). These eight significantly up-regulated miRNAs were hsa-let-7a-5p, hsa-let-7c-5p, hsa-let-7f-5p, hsa-miR-192-5p, hsa-miR-10a-5p, hsa-miR-10b-5p, hsa-miR-375, and hsa-miR-143-3p (Table 2). There was no significantly down-regulated miRNAs identified in NTG patients compared to control subjects according to the same significance criteria.

**Table 2:** Differentially expressed miRNA in the aqueous humor of normal tension glaucoma patients.

miRNA	Assay ID	Accession number	Fold change (Log2)	*p*-value	Expression change
			NTG/Control	NTG/Control	
hsa-let-7a-5p	1	MIMAT0000062	1.33	0.035	Up
hsa-let-7c-5p	3	MIMAT0000064	2.92	0.004	Up
hsa-let-7f-5p	6	MIMAT0000067	2.33	0.046	Up
hsa-miR-192-5p	46	MIMAT0000222	3.02	0.003	Up
hsa-miR-10a-5p	59	MIMAT0000253	4.85	0.001	Up
hsa-miR-10b-5p	60	MIMAT0000254	7.98	0.008	Up
hsa-miR-375	166	MI0000783	8.19	0.000	Up
hsa-miR-143-3p	1822	MIMAT0000435	1.74	0.037	Up

*NTG* normal-tension glaucoma.

### microRNA validation and biological interpretation of differentially expressed miRNAs

**Figure 2:**
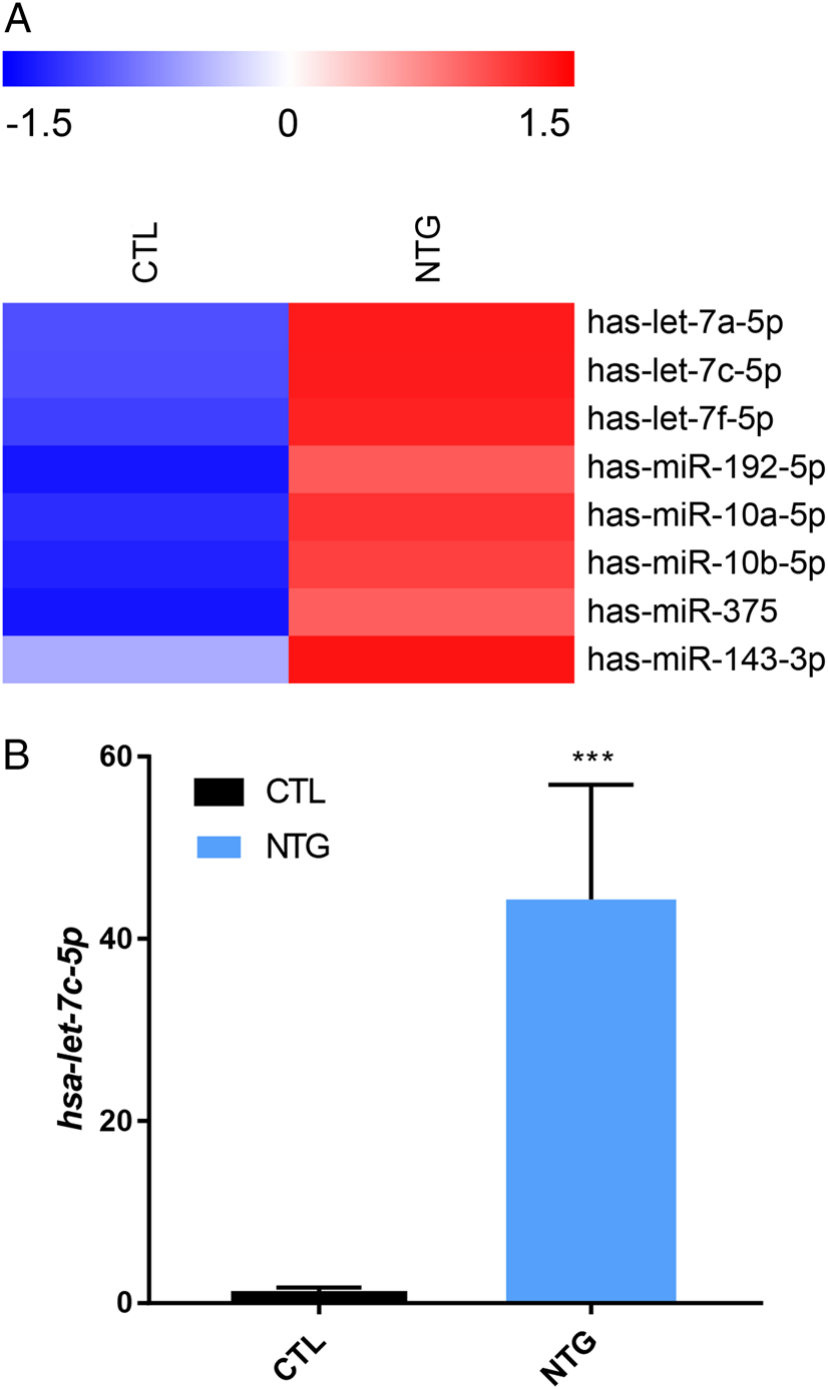
Quantitative PCR validation of miRNA in AH of NTG patients compared with control. (**A**) The heatmap diagram demonstrated the results of RNA sequencing by expression of miRNAs. Red represents high relative expression and blue represents low relative expression (Z-score). Data was presented by ExDEGA v1.2.1.0 software. (**B**) hsa-let-7c-5p expression in the AH of NTG patients by quantitative PCR validation analysis. The expression of let-7c-5p increased significantly in the AH of the NTG patients compared to control (44.32 ± 3.63-fold, *p* < 0.001). Data are represented as the mean ± S.E.M. and were analyzed using Unpaired Student’s t-test (n = 7). ***p < 0.001 versus Control. *AH* aqueous humor, *NTG* normal tension glaucoma, *S.E.M*. standard error of the mean.

RNA sequencing revealed eight significantly differentially expressed miRNAs between AH of NTG patients and control. The heatmap diagram shows those 8 miRNAs increased in AH of NTG patients (red, high relative expression, Z-score) compared to control (blue, low relative expression, Z-score) (Fig. 2A). To verify the results of RNA sequencing from the AH of NTG patients, let-7c-5p was analyzed using quantitative PCR (qPCR) (n = 7), and similar results were acquired (Table 2). The expression of let-7c-5p increased significantly in the AH of the NTG patients compared to control (44.32 ± 3.63-fold, *p* < 0.001) (Fig. 2B).

**Figure 3:**
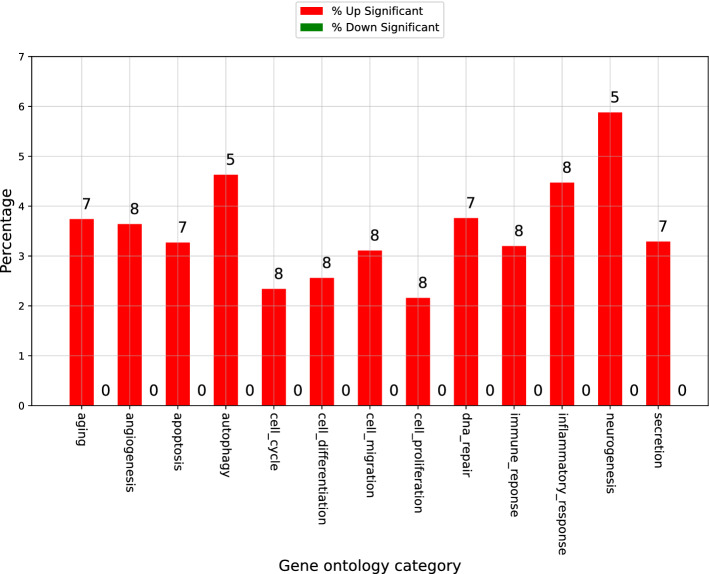
Percentage and number of microRNAs with significantly changed expression among gene ontology category-related microRNAs. Gene ontology (GO) category of microRNAs with relatively large expression changes are identified. Up-regulated miRNAs are shown as red graph and down-regulated miRNAs are shown as green graph. Cell death-related categories including apoptosis (3.27%) and autophagy (4.63%) occupied the major proportion. Categories related to neurogenesis (5.88%) and inflammatory response (4.47%) also mainly occupied the proportion. The percentage refers to the proportion of microRNA modified in AH of NTG patients compared to control in the total miRNAs identified by researches in each GO category. As significantly down-regulated miRNAs were not identified according to the same significance criteria with up-regulation, no green graph as down-regulated GO category is shown in this figure. Graphs were presented by ExDEGA v1.2.1.0 software.

To explore the effects of the expressed miRNAs, gene ontology (GO) categories were used. Fifteen major GO categories were randomly selected among numerous GO pathways. The percentage of the total significant and number of genes with differences in expression among each GO-related gene are presented in Fig. 3. The percentage refers to the proportion of microRNA modified in AH of NTG patients compared to control in the total miRNAs identified by researches in each GO category. Cell death-related categories, including apoptosis (3.27%) and autophagy (4.63%), occupied the major proportion. Categories related to neurogenesis (5.88%) and the inflammatory response (4.47%) also represented significant proportions. Categories related to cellular function, including proliferation, migration, and differentiation, which may occur in any pathological condition, occupied 7.83%. Those microRNAs involved in all of the gene ontology categories associated with biological processes of apoptosis, autophagy, and neurogenesis were hsa-let-7c-5p and hsa-miR-375. As significantly down-regulated miRNAs were not identified according to the same significance criteria with up-regulation (Fig. 1B), no green graph as down-regulated GO category is shown in Fig. 3.

**Figure 4:**
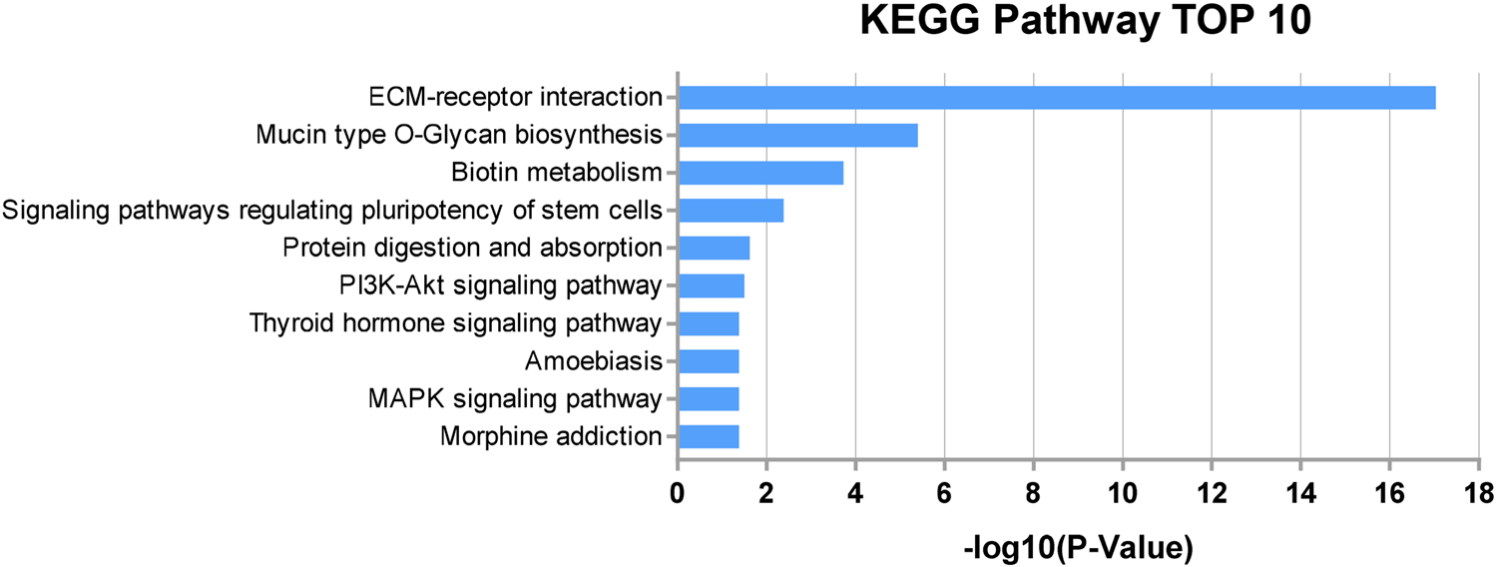
KEGG pathway. Enrichment score was represented as − log10 (*p*-value). The higher the enrichment score value is, the more significant the pathway is. The KEGG pathways relating to ECM-receptor interaction (hsa04512; 17.03), mucin-type O-glycan biosynthesis (hsa00512; 5.40), biotin metabolism (hsa00780; 3.73) and signaling pathways regulating pluripotency of stem cells (hsa04550; 2.39) were significantly associated with miRNAs increased in the aqueous humor of NTG patients. Note that the pathway relating to ECM-receptor interaction showed the most significant association with up-regulated miRNAs in NTG patients compared to other pathways. Data was analyzed by DianaTools. *KEGG* Kyoto Encyclopedia Genes and Genomes, *ECM* extracellular matrix.

The leading Kyoto encyclopedia of genes and genomes (KEGG) pathways, which include the predicted gene targets of each miRNA, are presented in Table 3. The analysis of gene-annotation enrichment was performed with the database for annotation, visualization, and integrated discovery (DAVID). The pathways relating to extracellular matrix (ECM)-receptor interaction (17.03, enrichment score, − log10 (*p*-value)), mucin-type O-glycan biosynthesis (5.40), biotin metabolism (3.73) and signaling pathways regulating pluripotency of stem cells (2.39) were significantly associated with miRNAs increased in the AH of the NTG patients. Among them, ECM-receptor interaction showed the most significantly related KEGG pathway in NTG (Fig. 4). The pathways such as protein digestion and absorption (1.62), and PI3K-Akt signaling (1.50) were also associated with up-regulated miRNAs.

**Table 3:** Significant KEGG pathways potentially influenced by microRNAs in the aqueous humor of normal tension glaucoma patients.

KEGG pathway	*p*-value (− log10)	Genes predicted as target	Related miRNAs
ECM-receptor interaction	17.027	18	6
Mucin type O-Glycan biosynthesis	5.402	7	6
Biotin metabolism	3.727	1	1
Signaling pathways regulating pluripotency of stem cells	2.387	31	8
Protein digestion and absorption	1.622	20	7
PI3K-Akt signaling pathway	1.505	58	8
Thyroid hormone signaling pathway	1.381	19	6
Amoebiasis	1.378	16	7
MAPK signaling pathway	1.378	42	8
Morphine addiction	1.378	16	8

*KEGG* Kyoto encyclopedia of genes and genomes, *NTG* normal-tension glaucoma, *ECM* extracellular matrix.

Since hsa-let-7c-5p and hsa-miR-375 were both involved in all of the gene ontology categories associated with biological processes of apoptosis, autophagy, and neurogenesis, we focused on these two microRNAs and inspected the KEGG pathways of each microRNA. The increase in hsa-let-7c-5p was the most associated with ECM-receptor interaction (18.1), like as shown in the results of KEGG pathway analysis in the AH of the NTG patients. Furthermore, the pathways such as amoebiasis (3.19), and mucin-type O-glycan biosynthesis (2.97) were also associated with hsa-let-7c-5p (Table 4). The increase in hsa-miR-375 was associated with biotin metabolism (8.34), the thyroid hormone signaling pathway (2.39), and the glutamatergic synapse (1.20) (Table 5).

**Table 4:** Significant KEGG pathways potentially influenced by up-regulated let-7c-5p in the aqueous humor of normal tension glaucoma patients.

miRNA	KEGG pathway	*p*-value (− log10)	Gene predicted as targets
hsa-let-7c-5p	ECM-receptor interaction	18.109	9
hsa-let-7c-5p	Amoebiasis	3.187	11
hsa-let-7c-5p	Mucin type O-Glycan biosynthesis	2.968	4
hsa-let-7c-5p	Glycosaminoglycan biosynthesis—chondroitin sulfate/dermatan sulfate	2.968	3
hsa-let-7c-5p	Signaling pathways regulating pluripotency of stem cells	2.422	16
hsa-let-7c-5p	PI3K-Akt signaling pathway	1.464	27
hsa-let-7c-5p	Protein digestion and absorption	1.464	11
hsa-let-7c-5p	Wnt signaling pathway	1.361	11

*KEGG* Kyoto encyclopedia of genes and genomes, *ECM* extracellular matrix.

**Table 5:** Significant KEGG pathways potentially influenced by up-regulated miR-375 in the aqueous humor of normal tension glaucoma patients.

miRNA	KEGG pathway	*p*-value (− log10)	Gene predicted as targets
hsa-miR-375	Biotin metabolism	8.340	1
hsa-miR-375	Thyroid hormone signaling pathway	2.388	7
hsa-miR-375	Glutamatergic synapse	1.997	8
hsa-miR-375	Protein processing in endoplasmic reticulum	1.753	13

*KEGG* Kyoto encyclopedia of genes and genomes.

## Discussion

To our knowledge, the present study was the first to report the microRNAs significantly differentially expressed in individual aqueous humor samples of NTG patients compared to controls in a single ethnic group of Asians. The present study did not pool the aqueous humor samples despite the scanty volume of each sample. RNA sequencing was performed to detect the microRNAs in each aqueous humor sample. RNA sequencing identified a total of 228 microRNAs in all aqueous humor samples. We detected 8 significantly upregulated microRNAs in NTG patients compared to the controls. They were hsa-let-7a-5p, hsa-let-7c-5p, hsa-let-7f-5p, hsa-miR-192-5p, hsa-miR-10a-5p, hsa-miR-10b-5p, hsa-miR-375, and hsa-miR-143-3p. Among them, the microRNAs involved in all the gene ontology categories associated with biological processes of apoptosis, autophagy, and neurogenesis were hsa-let-7c-5p and hsa-miR-375. Significantly downregulated microRNA was not identified in the present study according to the same significance criteria with up-regulation, although we also analyzed for downregulation in the process of RNA sequencing.

miRNAs have a significant part in the post-transcriptional modulation of gene expression and also are concerned in cellular functions including differentiation, growth, and cell death^27^. The human genome has approximately 2500 miRNAs that modulate the level of expression of > 60% of all genes that code proteins^19,28^.

Hsa-let-7c-5p miRNA is placed on human chromosome 21q21.1 on the LINC00478 gene. It has decreased expression and functions as a tumor suppressor gene for many malignancies such as colorectal adenocarcinoma, prostate, and acute myeloid leukemia^29,30^.

It has been reported that let-7 was downregulated in several malignancies and regulated apoptosis through direct targeting the oncogenes Myc, Ras, HMGA2, CDK6, and CDC25A^31^. MicroRNA let-7c has been reported to have an apoptotic effect on endothelial cells by suppressing anti-apoptotic protein Bcl-xl^32^. As one of the let-7 family, let-7a-5p has been reported to crosstalk with BCL-xL and lead to cell death and toxic autophagy in A549 lung cancer cells via activating the PI3K-signaling pathway, independent from apoptosis^33^. Injury to RGCs leads to glaucoma and the subsequent characteristic clinical findings of cupping in the optic disc and loss of visual field. The results of our study suggest a possible role for hsa-let-7c-5p in the pathogenesis of NTG on apoptosis and autophagy, which has not been reported before.

Hsa-miR-375 was the microRNA most significantly differentially expressed in the NTG patients compared to controls among all 13 aqueous humor samples (fold-change (log2) = 8.19, *p* = 0.000). Hsa-miR-375 was originally detected to be profusely expressed in isles of pancreas, and have a practical role in modulating glucose-stimulated insulin exocytosis^34,35^. In a study regarding mouse hippocampus, hsa-miR-375 was shown to affect dendrite formation^36^.

In a previous study, hsa-miR-375 was demonstrated to regulate neuronal cell death induced by ethanol^37^. Moreover, it was revealed that miR-375 upregulation was related with shrinkage of brain and defects in cognition in chronic alcoholism^37^. In another previous work, ketamine induced upregulation of hsa-miR-375 in hESC-derived neurons and it was concentration dependent^38^. The combined results of these studies^37,38^ suggest that miR-375 upregulation is associated with pathological cortical neuron environments. Several previous studies have shown the neuronal function of hsa-miR-375, including effects on dendrite formation^36^ and the modulation of neuronal apoptosis^39^. Previous work demonstrated that hsa-miR-375 inhibition showed epigenetic protection in hESC-derived neurons from ketamine induced neurotoxicity, probably via the direct and inverse modulation of the brain-derived neurotrophic factor (*BDNF*) gene^38^.

In a previous study, 11% of aged marmosets demonstrated NTG-like degeneration of the optic disc, which was a similar rate with that of glaucoma patients. The marmosets did not show elevated IOP but demonstrated elevated oxidative stress levels, low BDNF and TrkB expression, and low cerebrospinal fluid (CSF) pressure in the retina, optic nerve head, and CSF^40^. The expression of BDNF and TrkB were decreased in the optic nerve and retina in glaucomatous marmosets, in accordance with the data assembled from observations of glaucoma patients^41^. Neurotrophins are one of therapeutic candidates for glaucoma and several studies have shown that BDNF eye drops rescued visual function in a mouse glaucoma model with high-IOP (DBA/2J mice)^42,43^. Considering the direct regulation of hsa-miR-375 effect on the *BDNF* gene, the results of our study may also support the *BDNF* gene as a potential target for glaucoma gene therapy, especially for NTG.

RGC dendrites are considered to respond and demonstrate compensatory changes after damage, for example, extending the dendritic perceptive fields and generating new branches^44–46^. A recent study by Park et al. reported that synaptic vesicle proteins in bipolar cells and RGCs were augmented following BDNF application in a chronic IOP elevation rat model^47^. In addition, BDNF improved the number of synapses connecting bipolar cells and the RGCs in the retina of glaucomatous change. The authors concluded that these findings concerning the ability of BDNF to encourage useful synaptic changes may assist in the development of neuroenhancement approaches for the treatment of disturbed synaptic function in glaucoma. Our results also suggest that dendrite formation and the related BDNF involvement could serve as novel treatment targets for glaucoma, especially for NTG, regarding the neuronal functions of hsa-miR-375. Moreover, RGC dendrite formation and the related BDNF action, which are gene ontology categories associated with neurogenesis, may be involved in the pathogenesis of NTG.

The inflammatory response was one of the gene ontology biologic process categories potentially affected by microRNAs in the aqueous humor of NTG patients (Fig. 3). The binding of hsa-miR-375 to toll-like receptor 4 (TLR4) was verified by a dual-luciferase reporter gene assay^48^. TLR4 activates nuclear factor-kappa B (NF-κB) through MyD88, stimulating the secretion of downstream inflammatory cytokines^49^. Studies of patients with glaucoma and glaucoma experimental models indicated that the inflammatory response is mediated partly by toll-like receptors (TLRs)^50^. Studies of anatomy have reported elevated levels of TLR2, TLR3, and TLR4 in microglia and astrocytes in the retina of glaucoma patients^51^. Another previous study demonstrated that the inhibition of astroglial NF-kB decreased the inflammatory conditions and enhanced the survival of RGCs following retinal ischemia^52^. The association between hsa-miR-375 and TLR and the inflammatory response may partially explain the results of our study in NTG patients.

Aging was also one of the gene ontology biological process categories potentially affected by microRNAs in the aqueous humor of NTG patients (Fig. 3). As glaucoma is an age-dependent disease and the prevalence of glaucoma increases with age as reported in many population-based studies worldwide and also in Asians^9^, the results of our study seem reasonable.

The other gene ontology categories of biologic processes affected by microRNA in the aqueous humor of NTG patients compared to control were cellular proliferation, migration, differentiation, and cycle, DNA repair, secretion, and angiogenesis. These categories may act in common in any pathological conditions and may not occur solely in glaucoma. In this regard, such categories are not discussed in detail in the present study.

Degeneration of optic nerve fiber originally takes place at the site of lamina cribrosa in glaucoma^53^. The lamina cribrosa is organized by the ECM and inactive astrocytes, and acts as a fibroelastic structure, offering biological and mechanical support for axons of optic nerve head^54,55^. Glaucoma brings about remodeling of ECM and the activation of astrocytes. Consequently, the reactive astrocytes express new ECM proteins, which are regarded to change its configuration or be neurotoxic to the RGCs^56^. A study that used bioinformatics analysis to detect differentially expressed glaucoma genes found that one of the most significantly enriched pathways was ECM‑receptor interaction^57^. This finding is consistent with the results of the current study, which displayed that the most significant related KEGG pathway was ECM‑receptor interaction in NTG patients (*p*-value (− log10) = 17.027). Compared to ECM‑receptor interaction, other KEGG pathways showed *p*-value (− log10) of less than 5.402 and the least three were 1.378 (Table 3 and Fig. 4).

When we analyzed the KEGG pathway of each differentially upregulated let-7c-5p and miR-375, the most significantly related KEGG pathway in let-7c-5p was ECM-receptor interaction (*p*-value (− log10) = 18.109) (Table 4), which is in accordance with the results of all of differentially expressed microRNAs. We also confirmed the significant relative expression of let-7c-5p compared to the controls by qPCR (Fig. 2B). These validation results of confirmation of let-7c-5p which showed the same most significantly related KEGG pathway indicate the reliability and validity of our RNA sequencing results.

The pathogenesis of NTG is still under debate and it is a particular issue because NTG develops even within normal-range IOP. Studies using OCT imaging devices have reported differences in lamina cribrosa thickness and curvature in NTG patients^58–60^. The thickness of the lamina cribrosa was thinner in NTG than in POAG patients^58^. Moreover, the lamina cribrosa was thinner in VF-affected unilateral NTG eyes than in the fellow normal eyes of unilateral NTG patients or normal eyes^59^. In unilateral NTG patients, eyes with glaucomatous damage showed more steeply curved lamina cribrosa than the fellow healthy eyes^60^. These findings suggest that the lamina cribrosa undergoes significant remodeling in NTG eyes. Considering that the lamina cribrosa is the primary site of glaucomatous injury and NTG results in the remodeling of the lamina cribrosa, which is organized by the ECM, our microRNA analysis results indicate the important role of ECM alterations in the pathogenesis of NTG.

The other KEGG pathways possibly affected by microRNAs in the aqueous humor of NTG patients in our study included mucin-type O-glycan biosynthesis, biotin metabolism, and signaling pathways regulating the pluripotency of stem cells. In this current study, several gene ontology biological process categories and related KEGG pathways were associated with the differentially expressed microRNAs in the aqueous humor of the NTG patients. Confirmation of individual biological processes or KEGG pathways associated with NTG needs further clinical and experimental studies. However, our study is unique in that significantly differentially expressed microRNAs were identified in the individual sample of aqueous humor of NTG patients compared to the controls in a single ethnic group of Asians.

Since our study is the first to report the differentially expressed microRNAs in NTG patients and therefore, there is not yet a previous study to compare the results from. But we can compare our results from previous POAG patients with elevated IOP. Recent review article that investigated the mechanisms of microRNA in POAG described that microRNAs were involved in the regulation of IOP and related to optic nerve damage factors such as mechanical stress, hypoxia and inflammation^61^. Those microRNAs in POAG are miR-29b^62,63^, miR-143/145^64,65^, miR-182^66,67^, miR-200 family^68,69^, miR-204^70,71^, miR-155^72,73^ and miR-146a^74,75^. These microRNAs investigated in the pathogenesis of POAG were not identified in the current study in NTG patients with normal range of IOP, which suggest that the differentially expressed microRNAs in the pathogenesis of NTG are different from POAG with high IOP. It may also partially indicate the reliability and validity of the present study to some extent.

The present exploratory study has limitations due to the relatively small sample size and the small volume of the aqueous humor samples, but it has a significant meaning in that it provides the potential for further research field of microRNA in NTG, which is prevalent in Asians. Since the volume of the aqueous humor samples was not enough for all miRNAs to undergo qPCR for validation, only hsa-let-7c-5p underwent qPCR, which showed a significant relative expression pattern consistent with the RNA sequencing results. Considering that hsa-let-7c-5p showed a much smaller fold-change (fold-change (log2) = 2.92) than that of hsa-miR-375 (fold-change (log2) = 8.19) in the NTG patients compared to the controls in RNA sequencing (Table 2), the consistent qPCR results (Fig. 2B) of hsa-let-7c-5p may indicate the reliability of our RNA sequencing results. The influence of hypotensive topical medications on microRNA expression within the aqueous humor of NTG patients has yet to be determined. The impact of using different hypotensive topical medications on our results is not known. Future studies with large numbers of samples would benefit from controlling the use of hypotensive topical medications.

In conclusion, we found significantly differentially expressed microRNAs in the individual aqueous humor of NTG patients compared to controls in a single ethnic group of Asians, which has not been investigated previously. The findings of the present study suggest a possible role of microRNA in the pathogenesis of NTG. Further studies with more cases should be performed to draw more definitive conclusions. In addition, microRNA in individual aqueous humor may have a further potential as biomarkers and novel targets for the pathogenesis of NTG.

## Material and methods

### Ethics statement

This study was conducted according to the tenets of the Declaration of Helsinki for research involving human subjects. The current study was approved by the Institutional Review Board of Gyeongsang National University Changwon Hospital, Gyeongsang National University, School of Medicine (GNUCH-2019-06-001-002). Written informed consent was obtained from all subjects enrolled in this study. All methods were carried out in accordance with relevant guidelines and regulations.

### Patient selection and acquisition of aqueous humor samples

Samples of aqueous humor were obtained from patients who underwent uneventful phacoemulsification for routine cataract surgery after obtaining written informed consent. Six patients with NTG stably managed with only topical medication and seven age-matched control subjects agreed to take part in the present study. Approximately 80 to 120 µl of aqueous humor was obtained by corneal paracentesis with a 30-gauge needle before the initial main cataract incision at the beginning of surgery. Anterior chamber paracentesis was performed in the operating room under aseptic sterile conditions. The collection of aqueous humor was performed without trauma in all subjects, thus, removing any possibility of contamination with cellular remains or blood. All of collected samples were completely anonymized and immediately snap-frozen with liquid nitrogen, then transferred to the research laboratories. The clinical data was obtained from the electronic medical record and collected in a completely anonymized way. The clinical data collected were age, sex, eye laterality of right or left, baseline IOP, topical eye drops used, and ocular comorbidities.

### RNA isolation

Total RNA was extracted using Trizol LS reagent (Invitrogen, Carlsbad, CA, USA) following the manufacturer’s instructions. The quality of RNA was evaluated by an Agilent 2100 bioanalyzer using the RNA 6000 Pico Chip (Agilent Technologies, Amstelveen, The Netherlands), and quantification of RNA was carried out employing a NanoDrop 2000 Spectrophotometer system (Thermo Fisher Scientific, Waltham, MA, USA).

### Library preparation and RNA sequencing

For the control and test RNAs, a library was constructed employing an NEBNext Multiplex Small RNA Library Prep kit (New England BioLabs, Inc., Ipswich, MA, USA) following the manufacturer’s instructions^76^. In brief, for library construction, 180 pg of total RNA from each sample was used to ligate 1ug of adaptors, and then cDNA was synthesized using reverse-transcriptase with adaptor-specific primers. PCR was performed for library amplification and the libraries were cleaned-up using a QIAquick PCR Purification Kit (Qiagen, Inc, Germany) and AMPure XP beads (Beckman Coulter, Inc., Pasadena, CA, USA). The yield and size distribution of the small RNA libraries were evaluated by the Agilent 2100 Bioanalyzer instrument for the High-sensitivity DNA Assay (Agilent Technologies, Inc., USA). High-throughput sequences were produced by the NextSeq500 system by single-end 75 sequencing (Illumina, San Diego, CA, USA).

### microRNA validation by quantitative real-time PCR

cDNA synthesis and real-time PCR were conducted employing the miScript PCR system (Qiagen, Venlo, The Netherlands). cDNA was synthesized from 357 pg of RNA employing the miScript II RT Kit with HiSpec buffer following the manufacturer’s instructions. The microRNA cDNA was amplified employing the following primer pair: hsa-let-7c-5p (Hs_let-7c_1, MS00003129) and internal control hsa-U6 (Hs_RNU6-2_11, MS00033740). Real-time PCR was conducted on a StepOnePlus Real-Time PCR System (Applied Biosystems, Foster City, CA, USA) using QuantiTect SYBR Green PCR Master mix and the miScript Primer Assay (Qiagen), following the manufacturer’s instructions. The thermal cycling conditions were 95 °C for 15 min followed by 40 cycles of 94 °C for 15 s, 55 °C for 30 s, and 70 °C for 30 s. The data analysis was performed employing StepOne software v2.2.2 (Applied Biosystems). The level of expression of each microRNA was normalized to the median Ct value and calculated using the 2^−ΔΔCt^ method.

### Data analysis

The sequence reads were mapped employing the bowtie2 software tool to acquire bam files (alignment file). The mature miRNA sequence was applied as a reference. The read counts mapped on mature miRNA sequences were extracted from the alignment file using bedtools (v2.25.0)^77^ and Bioconductor (EdgeR package) that employs R statistical programming language (R Development Core Team, 2011, version 3.2.2). Read counts were applied to detect the level of the miRNAs expression. For quality criteria, trimming was performed with BBDuk tool. Illumina Truseq adapter was used and phred quality threshold were over 20. The quantile normalization method was applied for the comparison between the samples. For the miRNA target study, DianaTools-mirPath v.3 (http://diana.imis.athena-innovation.gr/DianaTools/index.php?r=site/page&view=software) was employed. DianaTools, miRTarBase (http://mirtarbase.mbc.nctu.edu.tw/php/search.php), miRWalk 2.0. (http://zmf.umm.uni-heidelberg.de/apps/zmf/mirwalk2/) and TargetScan (http://www.targetscan.org/vert_72/) were used to predict the miRNA targets. Related KEGG pathways were analyzed in accordance with previous studies from Kanehisa Laboratories^78–81^. Data were presented with ExDEGA v1.2.1.0 software (EBIOGEN, Inc., Seoul, Korea).

### Statistical analysis

Enrichment *p*-values were corrected for false discovery rate (FDR)^82^. Two sample t-test was employed for quantitative PCR validation. Data of microRNA validation are expressed as the mean ± standard error of the mean (S.E.M.). Statistical significance for the validation was determined using Unpaired Student’s t-test (Prism 5; GraphPad Software, La Jolla, CA, USA). *p* < 0.05 was considered to indicate a statistically significant difference.
